# The Influence of Playing Position and Contextual Factors on Soccer Players’ Match Differential Ratings of Perceived Exertion: A Preliminary Investigation

**DOI:** 10.3390/sports6010013

**Published:** 2018-02-12

**Authors:** Steve Barrett, Shaun McLaren, Iain Spears, Patrick Ward, Matthew Weston

**Affiliations:** 1 Sports Medicine and Science Department, Hull City FC, Kingston Upon Hull HU16 4HB, UK; 2 Sport Science and Medical Department, Hartlepool United Football Club, Hartlepool TS24 8BZ, UK; S.Mclaren@tees.ac.uk; 3 Department of Psychology, Sport and Exercise, School of Social Sciences, Humanities and Law, Teesside University, Middlesbrough TS1 3BX, UK; M.Weston@tees.ac.uk; 4 Pro-Football Support, Huddersfield HD7 5BQ, UK; iain.spears@gmail.com; 5 Research Institute for Sport and Exercise Sciences, Liverpool John Moores University, Liverpool L3 5UA, UK; pward2@gmail.com

**Keywords:** training load, RPE, monitoring

## Abstract

(1) Background: Differential RPE (dRPE) separates scores for breathlessness (RPE-B), leg muscle exertion (RPE-L) and technical/cognitive exertion (RPE-T). Limited information for dRPE is available in soccer match play, yet these measurements may help inform practitioners training and recovery strategies. This preliminary study investigated the effects of playing position and contextual factors on elite soccer players’ dRPE. (2) Methods: Thirty-two male English Premier League players recorded dRPE scores 15–30 min post-match for RPE-B, RPE-L, and RPE-T. Data were analysed using linear mixed models, with magnitude-based inferences subsequently applied. (3) Results: Overall, the mean ± SD for the dRPE were 63 ± 23 arbitrary units (au) (RPE-B), 67 ± 22 au (RPE-L), and 60 ± 24 au (RPE-T). Full Backs reported substantially higher RPE-B, RPE-L and RPE-T when compared to all other positions. Substantially higher RPE-T scores were reported for matches played against Top teams compared to Bottom (10 au; 90% Confidence Interval 5 to 15 au) and Middle (10 au; 4 to 15 au) ranked teams. The effects of match result and location on dRPE were not substantial. (4) Conclusions: Positional differences were observed following soccer match play for RPE-B, RPE-L and RPE-T. Full backs had substantially higher dRPE then any other position, with all players reporting increased RPE-T when playing teams at the Top of the league. These findings can help practitioners monitor internal load responses and support the prescription of training and recovery sessions.

## 1. Introduction

Soccer match play represents a complex interaction of physical and technical activities, which can take between 48 and 72 h to recover back to baseline levels [1,2]. When these demands are combined with playing duration, matches represent a large percentage of players’ load during the competitive season [3]. Technology-based activity measures, such as semi-automated match analysis and global positioning satellite systems, can now provide scientists and practitioners with detailed information on players’ match loads, such as distances covered and number of sprints. However, for an accurate prescription of post-match recovery and training strategies, scientists and practitioners require an understanding of a player’s internal response to match play [4]. Session ratings of perceived exertion (sRPE) are widely-used measures of internal match load, yet a global sRPE score may lack sensitivity [4] to account for the highly variable external loads observed in soccer match play [5]. In this context, differential ratings of perceived exertion (dRPE)—separate scores for breathlessness, leg muscle exertion and technical/cognitive exertion [4]—may be a viable alternative for the measurement of internal loads [4,6,7].

Understanding a player’s internal response to external match loads can help practitioners recommend appropriate recovery strategies, based upon the potential onset of fatigue induced from match play. However, there are several match contextual factors that impact on the physical, technical and tactical demands placed on an individual player, with playing position [5], match scoreline [8,9], match location [10] and opposition standard [11,12], all influencing external load. For example, Full Backs, Centre Midfielders and Wide Midfielders have higher volumes and intensities of match running when compared to Centre Defenders and Attackers [5,12]; players cover more total distance for matches won compared to matches drawn and lost [8,9]; and greater low-intensity running distances are associated with playing at home [8]. Furthermore, when playing against a higher standard of the opposition, lower-ranked teams have been shown to have greater locomotor activities, such as total distance covered and distances covered at high speed thresholds (>5.5 m/s), with a reduction in technical and possession statistics [13].

To the authors’ knowledge, limited information is available examining the internal match loads, and more specifically how the positional and contextual factors associated with match play effect the internal responses. Therefore, our aim here was to undertake a preliminary investigation into the effects of playing position and contextual factors on elite soccer players’ internal match loads, as measured via differential ratings of perceived exertion.

## 2. Materials and Methods

### 2.1. Subjects

Thirty-two male, professional, soccer players (Age: 25 ± 8 years; Stature: 1.80 ± 0.09 m; Body Mass: 85.2 ± 8.6 kg) from the same English Premier League team (top tier of English soccer) were initially included in the study. Players were observed across the duration of the 2016/2017 English FA Premier League soccer season (August to May). The ethics committee of the School of Social Sciences, Humanities and Law at Teesside University approved the study, which conformed to the Declaration of Helsinki.

### 2.2. Procedures

#### 2.2.1. Collection of dRPE

Between 15- and 30-min post-match, players recorded match dRPE sores for breathlessness (RPE-B), leg muscle exertion (RPE-L), and technical demand (RPE-T). Scores were recorded individually using a numerically blinded CR100^®^ scale [14] via a custom-built mobile application running on a 7″ Android tablet (Iconia One 7 BI-750, Acer Inc., Taipei, Taiwan) [15]. The CR100^®^ scale was chosen over the more commonly used CR10^®^ RPE as the scales finer grading has potential to provide a more sensitive appraisal of exertion in soccer players [16]. Each player was familiar with the scale, and the recommended researcher instructions for scale administration were used [17]. Specifically, the players were prompted with the following screen text for each dRPE measurement: “Using the verbal expressions on the scale below, please rate your (individual dRPE measure) perception of exertion for the match”. Each dRPE measurement was individually shown on the screen, with a sliding scale to mark the appropriate word/line to describe their exertion.

#### 2.2.2. Classification of Playing Position and Match Contextual Factors

Playing position was categorised as Goalkeeper, Full Back, Central Defender, Wide Midfielder, Central Midfielder, and Attacker, as per recent work [18]. Match result (Win, Draw, Loss) and location (Home, Away) were recorded and retained by the first author of the study. A retrospective ranking of match opposition standard was classified as per the team’s final league standing (Top, 1st to 6th; Middle 7th to 13th; Bottom, 14th to 20th) [10,19]. While we acknowledge that this classification may lack temporal sensitivity [20], the final standing after an entire season (i.e., 38 matches) clearly reflects a teams’ ability.

#### 2.2.3. Data Reduction

Given the preliminary nature of our study, we applied a stringent inclusion criterion. Players were only included in the final analysis if they had provided dRPE data from a minimum of three complete matches (i.e., 90 min). Goalkeepers were excluded. In total, 546 individual match observations were recorded from 29 players prior to the inclusion criteria. The inclusion criteria resulted in a total of 217 match observations (range 3 to 25) from 18 players. Some players (*n* = 7) meeting the inclusion criteria played in more than one of the five playing positions, as four players played as a Wide (range 1 to 10 matches) and Central Midfielder (1 to 21), one player Full Back (24 matches) and Wide Midfielder (1), one player Full Back (1) and Central Defender (14), and one player Full Back (5), Central Defender (1) and Central Midfielder (8). The final dataset was comprised of: Full Backs (Player *n* = 3, Match *n* = 30); Central Defenders (*n* = 5, *n* = 57); Wide Midfielders (*n* = 8, *n* = 71); Central Midfielders (*n* = 7, *n* = 44); Attackers (*n* = 3, *n* = 15).

### 2.3. Data Analysis

Given that elite soccer players’ physical and technical match profiles are influenced by playing position [21] and contextual factors such as match result, location, opposition standard [20], we employed mixed linear modelling (SPSS v24, IBM Corp, Armonk, NY, USA) to determine any such effects on players’ dRPE scores. Using a reduction of >10 in the Bayesian Schwartz Criterion as reference [22], an iterative, stepwise process was used to select the best fit model, from a basic model to one that included fixed and random effects and intercepts. For all dRPE, the final models were represented by four match contextual factors (playing position, result, location, opposition standard) as fixed effects and player included as a random effect with a random intercept to account for the hierarchical research design (i.e., repeated matches nested within player). The magnitude of all fixed effects was interpreted using a threshold of 10% of the dRPE score (6 au for RPE-B and RPE-T, and 7 au for RPE-L), as this value represents a likely substantial between-match change in dRPE [4]. From here, t statistics were created for all comparisons (mean difference—threshold/standard error of the difference), which were then converted to a probability via the t-distribution. These probabilities were then interpreted using magnitude-based inferences [23]. Given the preliminary nature of our study, combined with the relatively low number of players, we adopted a conservative approach to inference whereby substantial effects were only declared clear when the probability likelihood for the effect was ≥75% (i.e., likely). Further, we elected not to perform any interactions of our fixed effects (e.g., location*opposition standard) as caution about any such effects would have been required given these analyses were not hypothesised in advance [24]. The precision of all estimates is presented as 90% confidence intervals and analyses were performed on the raw untransformed data given that visual inspection of residual plots revealed no evidence of heteroscedasticity.

## 3. Results

Overall, the mean ± SD for the dRPE were 63 ± 23 arbitrary units (au) (RPE-B), 67 ± 22 au (RPE-L), and 60 ± 24 au (RPE-T). The teams match results were 31% Win, 19% Draw, and 50% Loss. Match location was evenly distributed between Home (49%) and Away (51%) matches, with the proportion of matches played against Top (36%), Middle (31%), and Bottom (33%) ranked opposition also evenly distributed. The teams final league position was 18 of 20. Presented in Table 1 are the dRPE scores for each of the four match contextual variables and their associated sub-levels.

### 3.1. RPE-B

Full Backs reported substantially higher RPE-B when compared to Central Defenders (21; 90% confidence interval 5 to 37), Attackers (24; 3 to 45), Wide Midfielders (13; −1 to 27), and Central Midfielders (12; −1 to 25). All remaining effects for playing position (range of the differences 1; −8 to 10–12; −7 to 31), along with effects for match result (0.2; −6 to 7–3.7; −7 to 7), match location (0.2; −5 to 6), and match opposition standard (0.9; −5 to 6–7.3; 2 to 13) were not substantial.

### 3.2. RPE-L

Substantially higher RPE-L were reported by Full Backs compared to Attackers (28; 90% confidence interval 12 to 46), Wide Midfielders (14; 8 to 28), and Central Defenders (15; 9 to 29) and also by Central Midfielders when compared to Attackers (17; 0 to 34). All remaining effects for playing position were not substantial (0; −13 to 13–13; −4 to 30), nor were the effects for match result (0.7; −6 to 7–2.7; −4 to 9) and match location (2.1; −3 to 7). Substantially higher RPE-L scores were reported for matches played against Top teams when compared to Bottom ranked teams (9; 4 to 15). The remaining effects for opposition standard were not substantial (1.7; −4 to 7 and 8; 2 to 13).

### 3.3. RPE-T

RPE-T were substantially higher for Full Backs compared to Central Midfielders (24; 90% confidence interval 11 to 37), Wide Midfielders (20; 7 to 34), Attackers (25; 3 to 47), and Central Defenders (19; 3 to 35). The remaining effects for playing position were not substantial (1; −19 to 21–6; −17 to 27), nor were the effects for match result (1; −4 to 7–3; −3 to 9) and match location (6; 1 to 11). Substantially higher RPE-T scores were reported for matches played against Top ranked teams when compared to Bottom (10; 5 to 15) and Middle (10; 4 to 15) ranked teams, with no difference in RPE-T between matches played against Middle or Bottom ranked teams (1; −5 to 6).

## 4. Discussion

Internal load variables can demonstrate a stimulus for both positive and negative outcomes within a training programme [25]. Differential ratings of perceived exertion have been proposed as being a worthwhile addition to internal load monitoring procedures in team sports [15], yet there remains a paucity of information regarding the initial quantification for interpreting internal match loads. Our study represents the first examination of soccer players’ match dRPE loads. We found that Full Backs reported higher dRPE scores when compared to all other playing positions and that playing against a higher-ranked opponent was associated with higher RPE-L and RPE-T.

Discriminating between different perceived internal load measures have provided a detailed profile of an individual during a given activity [6]. Using specific physiological mediators to differentiate internal load responses has provided evidence that athletes responses are task specific [4,6]. However, soccer match play encompasses a variety of different physical, technical and tactical requirements for players to perform at an elite level, depending on their position [5]. A soccer player’s response to these physiological mediators can help practitioners understand the response to the match and consequently prescribe appropriate methods to support the recovery process [26]. Our study demonstrates mean match RPE-B and RPE-L scores that were consistent (e.g., very hard) with those reported previously in soccer [27] yet substantially lower than scores reported for AFL (Australian Football League) match play. This discrepancy is maybe attributable to the higher external loads associated with AFL match play (e.g., total distance ~13 km) [4]. While it was beyond the scope of the current study, future research should look at examining the external training load measures from soccer match play/training to further understand what influences dRPE.

Soccer players physical output from match play has shown to vary depending on match contextual variables and playing position, which may influence how practitioners plan for training and recovery sessions. Contextual factors observed to influence a player’s response involve both team (league positon, standard of opposition, home or away fixture) [12] and individual-based variables (position, age/years of experience, international/non-international player, physical fitness) [28]. It has been recommended that soccer players’ positions should be accounted for within research due to the large differences and variability within each positions locomotor activities [5,21]. In the current study, Full Backs reported substantially higher RPE-B, RPE-L, and RPE-T in comparison to all other playing positions. Carling and colleagues [29] reported that Full Backs performed the highest number of repeated high speed bouts during actual match play, which may be reflective of the dRPE scores reported in the current study. While it is acknowledged that Full Backs may not perform the highest distance covered at high speed or sprinting thresholds [5,29,30], performing multiple efforts within a short period of time may induce greater levels of acute fatigue within match play [31], with potential impact on a player’s subsequent speed of recovery over the proceeding 48–72 h period [1]. Further research is warranted to examine what external load variables directly influence RPE-B and RPE-L within soccer specific activities.

The physical and technical/tactical elements of soccer match play tend to be measured and analysed separately, despite one potentially influencing the other [12]. Russel and colleagues [13] found that similar decrements in player’s technical success and amount of possession was observed toward the end of each half of match play, similar to that shown by external load variables [5,32]. To the authors’ knowledge, no research is available examining a player’s perceived technical exertion from soccer match play. A novel finding of our work, therefore, was that players reported the match technical demand to be ‘very hard’. While exploratory, these initial findings suggest that some players perceive the technical/cognitive elements of the game as hard as their physical perceptions, or in some individual cases (See Table 1), even harder. To date, the only previous collection of this variable was for AFL, where the scores were much higher. Again, this may be reflective of match play technical differences between soccer and AFL [4].

With RPE-T in soccer and other team sports, there are a number of other confounding factors that may influence the individual’s level of perceived exertion. For example, the standard of the opposition (top 6 team vs. bottom 6 team) may have implications to the tactics employed by the manager/coaching staff, which has been demonstrated to influence the physical profiles of soccer players in the English Premier League [12]. Within the current study, players reported substantially higher RPE-T when they played against the Top teams within the division, which may be suggestive of increased mental/technical requirements of the players during these games. However, teams within the Bottom part of the league have been reported as having a lower amount of technical actions (i.e., number of passes, shots on goal) and amount of possession against opponents in the Top section of the league [11]. Further investigation into what influences and potentially explains RPE-T is required to better understand how and if this helps inform practitioners of either mental or physical fatigue. For example, a forward player may perceive a greater level of RPE-T based upon the number of goal scoring opportunities within a game, or a defender may be influenced by the number of times an opponent attacks the defensive third/the quality of the opposition forward. High cognitive loading has shown to induce feelings of tiredness, resulting in the reduction of maximal Electromyography (EMG) activity, something which requires further investigation within soccer and the potential implications of this with regards to appropriate recovery strategies [1]. Not only may these findings help practitioners within their monitoring of players recovery and fatigue status, it may also inform coaches of certain activities to work on with individual players to improve their technical/tactical understanding of the game. Finally, and in agreement with previous work [33], we encourage practitioners and soccer coaches to work closely together when interpreting load monitoring data to ensure practices are indeed player-focused.

## 5. Conclusions

Positional differences were observed during soccer player across RPE-B, RPE-L and RPE-T, with Full Backs reporting substantially higher dRPE than any other position. Players reported higher RPE-T when playing teams at the Top of the league, a novel finding in soccer match play. Taken together, these findings show potential for dRPE data collection as a practical method of monitoring internal load during elite soccer match play. Furthermore, dRPE can help practitioners better understand the internal load responses of their players, which may help support with the prescription of both training and recovery sessions. 

## Figures and Tables

**Table 1:** Estimated marginal means ± SD derived from the mixed linear model for each fixed effect (playing position, result, location, and opposition standard).

Match Contextual Variable	dRPE		
	RPE-B (au)	RPE-L (au)	RPE-T (au)
*Playing Position*			
Full Backs	78 ± 22 *	80 ± 21 *	81 ± 22 *
Central Defenders	57 ± 16	65 ± 14	62 ± 17
Wide Midfielders	65 ± 16	66 ± 14	61 ± 17
Central Midfielders	67 ± 15	69 ± 13 ^$^	57 ± 15
Attackers	55 ± 17	53 ± 15	56 ± 18
*Match Result*			
Win	66 ± 24	68 ± 23	62 ± 22
Draw	66 ± 27	66 ± 25	63 ± 24
Lose	62 ± 20	65 ± 17	65 ± 18
*Match Location*			
Home	64 ± 19	68 ± 17	66 ± 18
Away	64 ± 21	66 ± 18	60 ± 19
*Match Opposition Standard*			
Top	69 ± 22	72 ± 20 ^#^	70 ± 20 ^#^
Middle	62 ± 22	65 ± 20	60 ± 20
Bottom	63 ± 21	63 ± 18	60 ± 19

SD, standard deviation; dRPE, differential ratings of perceived exertion; RPE-B, ratings of perceived breathlessness, RPE-L, ratings of perceived leg muscle exertion, RPE-T, ratings of perceived technical exertion; au, arbitrary units; *, substantially higher than all other Playing Positions; ^$^, substantially higher than Attackers; ^#^, substantially higher than Middle and Bottom.
